# Prevalence and Associated Risk Factors of Tandem Spinal Stenosis

**DOI:** 10.7759/cureus.118012

**Published:** 2026-10-16

**Authors:** Mariam M Ali, Mohamed State, Ahmed Abdelkhalek, Ashraf S Ziden, Hanee Ali

**Affiliations:** 1 Neurosurgery Department, Faculty of Medicine, Mansoura University, Mansoura, EGY; 2 Diagnostic Radiology Department, Faculty of Medicine, Mansoura University, Mansoura, EGY

**Keywords:** cervico-lumbar stenosis, cross-sectional study, lee classification, predictive factors, prevalence, spinal stenosis, tandem spinal stenosis, whole-spine mri

## Abstract

Background: Tandem spinal stenosis (TSS) is a complex, multi-level degenerative condition that is notoriously underdiagnosed due to overlapping symptoms and diagnostic masking. The true prevalence and predictive risk factors of this condition remain inadequately characterized in patients presenting with focal spinal symptoms.

Objective: This study aimed to determine the prevalence of TSS and identify its associated demographic, anthropometric, clinical, and radiological factors in symptomatic patients.

Methods: A cross-sectional study was conducted at a tertiary neurosurgical center. Patients with symptomatic spinal stenosis were consecutively enrolled. Those with prior spinal surgery, MRI contraindications, or unrelated neurological deficits were excluded. Data collected included sociodemographic variables, anthropometrics, symptom duration, and comorbidities. All participants underwent whole-spine MRI, evaluated using the Lee grading system (0-3). TSS was defined as stenosis (Lee Grade ≥ 1) in ≥2 non-contiguous spinal regions, with a pre-specified sensitivity analysis using Grade ≥ 2. Predictors were identified using bivariate and binary logistic regression analyses.

Results: Between March 2025 and January 2026, 226 patients were enrolled (mean age 56.3 ± 13.9 years; 54.0% female). Overall, TSS prevalence was 80.1% (181/226; 95% confidence interval (CI): 74.9%-85.3%), with cervico-lumbar TSS as the dominant subtype (97.2%). A pre-specified sensitivity analysis (Lee Grade ≥ 2) yielded a substantially lower TSS prevalence of 45.6% (103/226; 95% CI: 39.1%-52.1%). In multivariate analysis, older age (adjusted odds ratio (aOR) 1.06, 95% CI: 1.03-1.08, p < 0.001) and longer leg pain duration (aOR 1.13, 95% CI: 1.04-1.23, p = 0.004) were independently associated with TSS.

Conclusions: TSS is highly prevalent (80.1%) among symptomatic stenosis patients. These findings suggest that a low threshold for whole-spine MRI may be clinically warranted in older patients presenting with chronic radicular symptoms to facilitate earlier detection and surgical planning.

## Introduction

Spinal stenosis is characterized by a reduction in the cross-sectional area of the spinal canal, leading to upper or lower motor neuron deficits and related neurological symptoms depending on the location of compression [1,2]. Spinal stenosis can affect more than one level in any segment [3]. When stenosis occurs simultaneously in two or more non-contiguous spinal regions, predominantly the cervical and lumbar spine, the condition is termed tandem spinal stenosis (TSS) [4-6]. TSS was first reported in 1957 by Brain and Wilkinson, who described a patient with concomitant cervical spinal stenosis (CSS) and lumbar spinal stenosis (LSS) [7]. Subsequently, Teng and Papatheodorou reported seven cases of concurrent CSS and LSS [7,8], and the term “tandem spinal stenosis” was formally introduced by Dagi et al., who defined it as a clinical triad of intermittent lower limb claudication, gait disorder, and combined upper and lower motor neuron signs [4]. TSS is now classified into four subtypes according to the stenotic regions involved: cervico-lumbar (CLTSS), cervico-thoracic (CTTSS), thoraco-lumbar (TLTSS), and cervico-thoraco-lumbar (CTLTSS) [9], with CLTSS being the most commonly encountered subtype, while thoracic vertebral involvement occurs with a comparatively low incidence [10].

As the global population ages, the burden of degenerative spinal diseases is steadily increasing, with a corresponding rise in TSS prevalence [11]. The reported incidence of radiological TSS ranges from 8% to 60%, while symptomatic TSS is estimated at 5%-28% [12]. In the general population, the incidence of CLTSS is 0.12%-11% [12]; however, among patients already diagnosed with CSS or LSS, the radiological incidence of coexisting CLTSS is substantially higher, ranging from 32.2% to 84.6% [12,13]. Degenerative spondylosis, driven by aging, lifestyle factors, and cumulative mechanical stress, is the primary etiology of CLTSS [12,14]. In subtypes involving the thoracic spine, heterotopic ossification, including ossification of the posterior longitudinal ligament (OPLL) and ossification of the ligamentum flavum (OLF), is a more prevalent etiological factor [12]. Developmental spinal stenosis (DSS) is a recognized risk factor, and the Torg-Pavlov rate (TPR), with a predictive threshold of less than 0.75-0.78, remains the only statistically significant predictor of coexisting stenosis reported in the literature [12,14]. Patients with stenosis in one region are also more likely to develop TSS; the presence of CSS positively predicts LSS in 16.7% of cases, and the presence of LSS predicts CSS in 15.3% of cases [12].

The clinical presentation of TSS is complex, as the coexistence of multi-level stenosis produces overlapping neurological findings that complicate accurate localization [15]. A hallmark feature is diagnostic “concealment,” whereby symptoms arising from one stenotic region can partly mask or obscure findings attributable to another; in particular, CSS may cover up symptoms of coexisting thoracic or lumbar stenosis, and lower motor neuron signs from lumbar compression may blunt the upper motor neuron findings of cervical cord involvement [15,16]. Diagnosing TSS remains a significant challenge, as the symptom profile is not simply the additive combination of individual stenotic segments [15]. Furthermore, standard imaging protocols frequently fail to encompass the entire spine, contributing to the missed identification of tandem lesions [17]. The missed diagnosis of tandem stenosis may result in prolonged preoperative duration and a poorer prognosis, as the underlying cause is inversely linked with outcomes [5,18]. Indeed, because of this diagnostic masking, TSS is notoriously underdiagnosed, with recent literature suggesting that up to 45% of cases are missed or diagnosed in a delayed fashion [19].

The treatment of TSS encompasses both non-surgical and surgical interventions. Existing evidence recommends that patients undergo two to three months of standardized non-surgical treatment before considering surgical intervention [11,20]. Non-surgical options include bed rest during the acute phase, oral non-steroidal anti-inflammatory drugs (NSAIDs), neurotrophic drugs, epidural steroid injections, supervised exercise, and physical therapy [11]. Non-surgical interventions can help alleviate symptoms and facilitate early spinal function training to maintain muscle strength and slow disease progression [11]. The combination of supervised exercise and manual therapy has been shown to modestly improve pain, symptom severity, and physical function, representing a low-cost and non-invasive treatment option for patients with mild symptoms [11]. When surgical treatment is indicated, the primary goal is to alleviate compression on the spinal cord and nerve roots, reconstruct vertebral stability, and improve clinical symptoms [1]. Surgical strategies include simultaneous surgery, staged surgery, and single-site surgery, each with varying applicability and clinical efficacy [12,21]. However, surgeons are frequently faced with the dilemma of determining optimal surgical sequencing, and despite extensive research, no consensus currently exists on the ideal surgical approach for TSS [17].

Despite its clinical significance, the epidemiology of TSS in populations presenting with symptomatic stenosis, particularly in the Middle East and African context, remains inadequately characterized. Identifying the demographic and clinical factors associated with TSS would enable timely whole-spine imaging, earlier diagnosis, and more optimal surgical planning. This study aimed to determine the prevalence of TSS and its subtypes, and to identify independent demographic, clinical, and radiological factors independently associated with TSS in patients with symptomatic spinal stenosis at a tertiary neurosurgical center.

## Materials and methods

Patient selection criteria

This cross-sectional study was conducted at the Department of Neurosurgery, Mansoura University Hospitals, a tertiary neurosurgical center in Mansoura, Egypt, between March 2025 and January 2026. Patients presenting with symptomatic spinal stenosis, including back or neck pain, with or without neurogenic claudication, radiculopathy, or myelopathy, were consecutively enrolled. Patients were excluded if they had (i) a pacemaker or implanted metal device precluding MRI, (ii) prior cervical or lumbar spinal surgery, (iii) a pre-existing neurological deficit unrelated to stenosis, or (iv) refusal to participate.

Ethical approval was granted by the Institutional Review Board (IRB) of Mansoura University, Faculty of Medicine (Code: MS.24.11.2995). We obtained written informed consent from all participants before enrolment. The study adhered to the principles of the Helsinki Declaration.

Data collection

Standardized clinical data were recorded for each patient, including sociodemographic variables (age, sex, educational level, and occupation) and anthropometric measurements (height, weight, and body mass index (BMI)). Clinical variables included presenting symptom profile (back pain, neck pain, leg pain, and arm pain), symptom duration in months, and documented comorbidities (diabetes mellitus, hypertension, osteoporosis, and rheumatological disorders).

Radiological assessment

All participants underwent whole-spine MRI. Spinal canal stenosis was graded at the cervical, thoracic, and lumbar levels using the Lee grading system: Grade 0 (no stenosis), Grade 1 (mild), Grade 2 (moderate), and Grade 3 (severe). Additional degenerative pathologies documented, including OPLL, ligamentum flavum hypertrophy (LFH), and disc herniation, were also recorded.

All MRI scans were independently evaluated and graded by two neurosurgeons with subspecialty experience in spinal surgery, each blinded to the patient's clinical symptom profile at the time of radiological assessment. The final stenosis grade at each spinal level was determined by consensus between the two assessors. TSS status for each spinal region was assigned based on the highest stenosis grade present at any disc or vertebral level within that region. Interobserver agreement for Lee grading classification was assessed using Cohen's kappa coefficient.

To rigorously evaluate disease burden, two diagnostic thresholds were established: (1) Primary analysis: TSS was defined as the presence of stenosis of any severity (Lee Grade ≥ 1) involving two or more non-contiguous spinal regions (cervical, thoracic, or lumbar). (2) Sensitivity analysis: A pre-specified sensitivity analysis was conducted restricting the definition to clinically substantial stenosis (Lee Grade ≥ 2, moderate-to-severe) in two or more non-contiguous spinal regions to prevent overestimation driven by mild degenerative changes.

Sample size calculation

The sample size was determined using Cochran’s formula for prevalence studies. Assuming an expected prevalence of TSS of 80% (based on hospital-based radiological prevalence reported) [12], and supported by pilot data from our center, with a 95% confidence level and a margin of error of 5.5%, the minimum required sample size was calculated to be 203 patients. To account for an anticipated 10% non-response rate, the final target sample size was set at 226 participants. This estimate reflects the expected prevalence in symptomatic neurosurgical patients, which is substantially higher than population-based MRI studies [10].

Statistical analysis

Data were analyzed using IBM SPSS Statistics (version 31.0.1.0, IBM Corporation, Armonk, NY, USA) and R (version 4.4.2, R Foundation for Statistical Computing, Vienna, Austria). Graphical plots and data visualizations were generated using Python (version 3.11; Matplotlib version 3.8.0 and Seaborn version 0.13.0 libraries). Normality was assessed with the Kolmogorov-Smirnov test. Continuous normally distributed variables are reported as mean ± standard deviation (SD); non-parametric variables as median (IQR). Categorical variables are expressed as frequency (n) and valid percentage (%). Symptom-duration variables were analyzed only in patients reporting the corresponding symptom; patients without a specific symptom were excluded from that variable's analysis.

Bivariate comparisons between TSS-positive and TSS-negative groups used independent samples t-test or the Mann-Whitney U test for continuous variables, and chi-squared or Fisher's exact test for categorical variables. Variables with p < 0.20 on bivariate analysis or those of particular clinical relevance to this study were entered into a binary logistic regression using backward stepwise elimination (removal criterion: p > 0.10). Statistical significance was set at p < 0.05.

## Results

Baseline demographic characteristics

A total of 226 patients with symptomatic spinal stenosis were enrolled. The baseline demographic, anthropometric, clinical, and comorbidity profile of the study cohort is summarized in Table 1. The mean age was 56.3 ± 13.9 years, with the majority of patients aged 50-65 years (40.7%). The study demonstrated near gender parity (female: 54.0%, male: 46.0%). The mean BMI was 30.13 ± 4.99 kg/m², with the majority classified as either overweight (36.7%) or obese (49.1%). Back pain was the most frequently reported symptom (61.9%), followed by leg pain (49.1%). Diabetes mellitus was the most prevalent comorbidity, affecting 39.4% of patients.

**Table 1:** Baseline Demographics, Anthropometrics, and Clinical Characteristics of the Study Population SD: standard deviation; BMI: body mass index

Variable	Category	Total (n (%) or mean ± SD)
Demographics		
Age (years)		56.32 ± 13.88
	<50 years	68 (30.1%)
	50-65 years	92 (40.7%)
	>65 years	66 (29.2%)
Sex	Female	122 (54.0%)
	Male	104 (46.0%)
Education level	Secondary school	90 (39.8%)
	College/university	86 (38.1%)
	Primary school	42 (18.6%)
	No formal education	8 (3.5%)
Occupation	Unemployed	72 (31.9%)
	Office work	53 (23.5%)
	Retired	52 (23.0%)
	Manual labor	49 (21.7%)
Anthropometrics		
Weight (kg)		88.16 ± 11.50
Height (m)		1.72 ± 0.08
BMI (kg/m²)		30.13 ± 4.99
	Normal	29 (12.8%)
	Overweight	83 (36.7%)
	Obese	111 (49.1%)
Clinical symptoms		
Back pain		140 (61.9%), 10.53 ± 7.19
Leg pain		111 (49.1%), 8.80 ± 4.39
Arm pain		93 (41.2%), 7.71 ± 3.92
Neck pain		70 (31.0%), 10.96 ± 7.91
Comorbidities		
Diabetes mellitus		89 (39.4%)
Rheumatological disorders		56 (24.8%)
Osteoporosis		48 (21.2%)
Hypertension		47 (20.8%)
Other diagnoses		27 (11.9%)
Tumors		14 (6.2%)

Radiological findings and prevalence of TSS

Whole-spine MRI grading using the Lee classification is presented in Table 2 and Figures 1A, 1B. Interobserver agreement for Lee grading classification was substantial (κ = 0.78). The cervical and lumbar regions were the predominant sites of stenotic disease. In the cervical spine, the majority of patients demonstrated moderate (Grade 2: 42.0%) or mild (Grade 1: 30.5%) stenosis, with severe-grade stenosis (Grade 3) observed in 15.9%. In the lumbar spine, moderate stenosis was similarly the most common grade (46.0%), followed by severe stenosis (27.4%). Thoracic involvement was negligible, with only five patients (2.2%) demonstrating mild stenosis and no cases of moderate or severe thoracic stenosis. Disc herniation was the most prevalent concurrent degenerative pathology, affecting 76.1% of patients at the lumbar level and 68.6% at the cervical level (Figure 1C).

**Table 2:** Radiological Stenosis Grading, Degenerative Pathologies, and TSS Prevalence OPLL: ossification of the posterior longitudinal ligament; TSS: tandem spinal stenosis; CI: confidence interval

Variables	Category	n (%)
A. Stenosis grading (Lee classification)		
Cervical	No stenosis	26 (11.5%)
	Mild (Grade 1)	69 (30.5%)
	Moderate (Grade 2)	95 (42.0%)
	Severe (Grade 3)	36 (15.9%)
Thoracic	No stenosis	221 (97.8%)
	Mild (Grade 1)	5 (2.2%)
	Moderate/severe	0 (0.0%)
Lumbar	No stenosis	25 (11.1%)
	Mild (Grade 1)	35 (15.5%)
	Moderate (Grade 2)	104 (46.0%)
	Severe (Grade 3)	62 (27.4%)
B. Degenerative pathologies	Cervical, n (%)	Lumbar, n (%)
OPLL	45 (19.9%)	32 (14.2%)
Ligamentum flavum hypertrophy	48 (21.2%)	112 (49.6%)
Disc herniation	155 (68.6%)	172 (76.1%)
C. TSS prevalence and subtypes		
Primary definition (Grade ≥ 1)	TSS present	181 (80.1% (95% CI: 74.9%-85.3%))
	TSS absent	45 (19.9%)
TSS subtype distribution (n = 181)	Cervico-lumbar (CLTSS)	176 (97.2%)
	Cervico-thoraco-lumbar (CTLTSS)	5 (2.8%)
	Cervico-thoracic (CTTSS)	0 (0.0%)
	Thoraco-lumbar (TLTSS)	0 (0.0%)
Sensitivity analysis (Grade ≥ 2)	TSS present	103 (45.6% (95% CI: 39.1%-52.1%))
	TSS absent	123 (54.4%)

**Figure 1:**
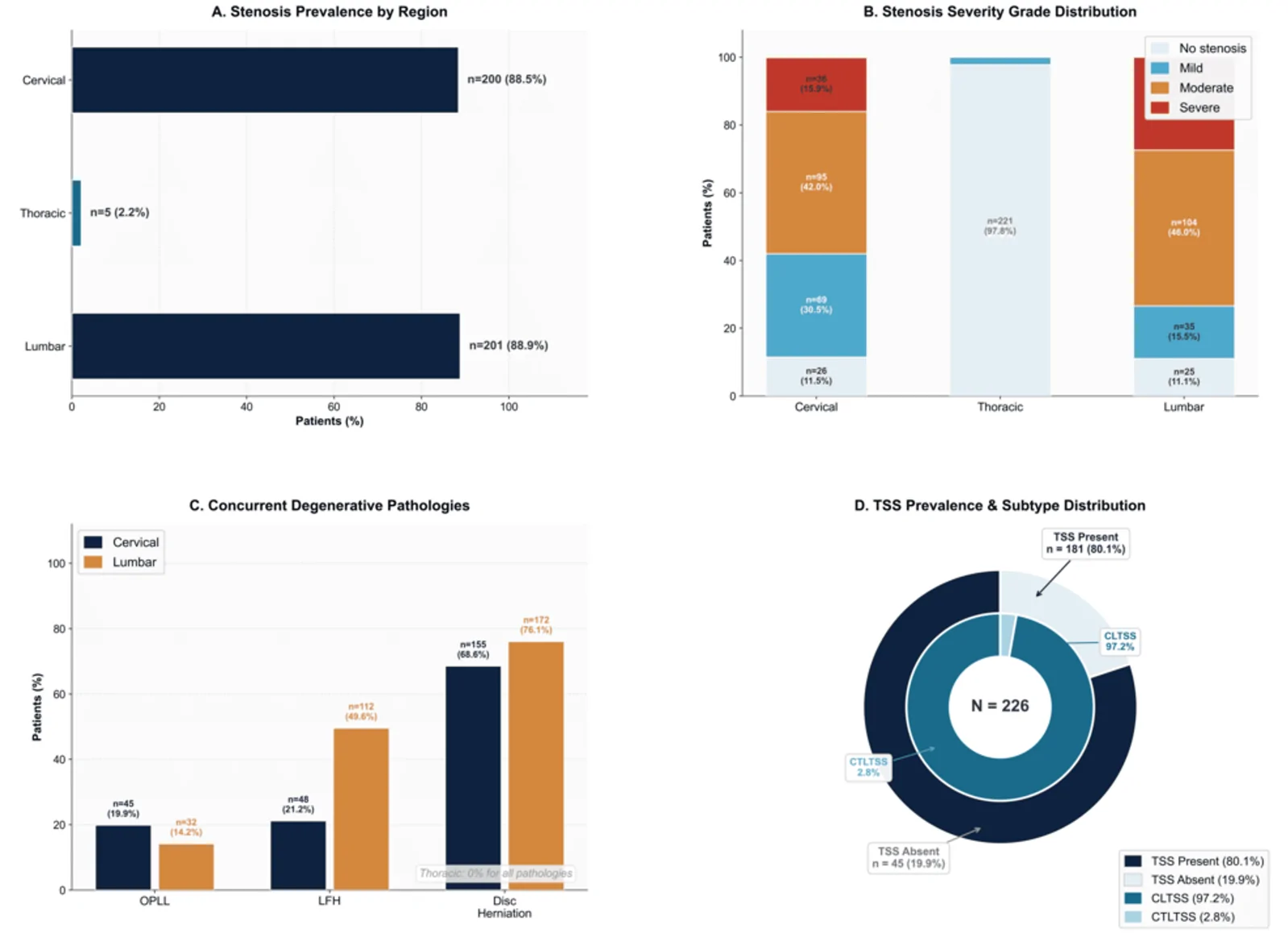
Radiological Profile and TSS Prevalence (N = 226) (A) Stenosis prevalence by region, (B) severity grade distribution, (C) concurrent degenerative pathologies, and (D) TSS prevalence and subtype distribution. Graphical panels were constructed from clinical MRI data using Python (version 3.11; libraries: Matplotlib 3.8.0 and Seaborn 0.13.0). TSS: tandem spinal stenosis; CLTSS: cervico-lumbar tandem spinal stenosis; CTLTSS: cervico-thoraco-lumbar tandem spinal stenosis; OPLL: ossification of the posterior longitudinal ligament; LFH: ligamentum flavum hypertrophy

In the primary analysis, TSS was defined as concomitant stenosis of any severity (Lee Grade ≥ 1, including mild) in ≥2 non-contiguous spinal regions. Using this definition, 181 of 226 patients (80.1%; 95% confidence interval (CI): 74.9%-85.3%) met the criterion for TSS (Figure 1D). CLTSS was the overwhelmingly dominant subtype, accounting for 97.2% of all TSS cases. Five cases of CTLTSS were identified (2.8%), while no CTTSS or TLTSS cases were observed. A pre-specified sensitivity analysis using a threshold of Lee Grade ≥ 2 (moderate-to-severe) in ≥2 non-contiguous regions yielded a substantially lower TSS prevalence of 45.6% (103/226; 95% CI: 39.1%-52.1%) (Table 2C).

To examine the anatomical correspondence between patient-reported pain symptoms and the documented site of stenosis, a cross-tabulation was performed mapping each primary pain symptom against the presence of radiologically confirmed stenosis at the cervical, thoracic, and lumbar spinal levels (Figure 2). Each cell displays the absolute count (n) and the column-normalized percentage (%) of stenotic patients at each level who reported the corresponding symptom. Back pain was the dominant symptom among both cervical stenosis patients and lumbar stenosis patients. Leg pain showed the highest association with lumbar stenosis, consistent with the expected neuroanatomical localization of lower-limb radiculopathy to lumbar-level compression.

**Figure 2:**
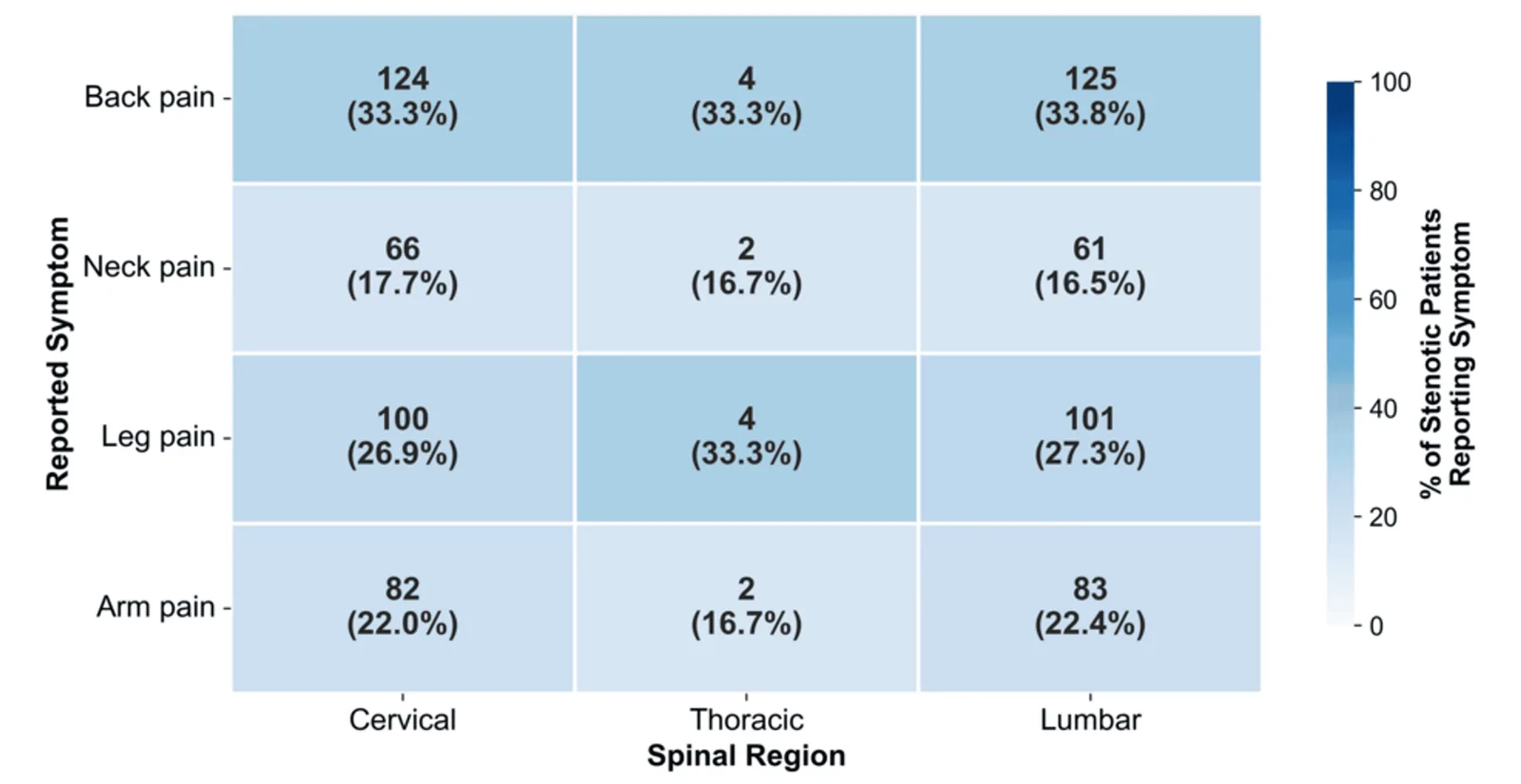
Symptom-Region Association Heatmap - Distribution of Reported Pain Among Patients With Stenosis at Each Spinal Level Cross-tabulation mapping patient-reported pain symptoms (back, neck, leg, and arm pain) against the anatomical site of radiologically confirmed stenosis on whole-spine MRI. Values in cells display absolute patient counts and column-normalized percentages. Heatmap visualization was plotted using Python (version 3.11; Seaborn library).

Binary logistic regression analysis: factors associated with TSS

On bivariate analysis (Table 3), TSS-positive patients were significantly older (58.29 ± 12.77 vs. 48.40 ± 15.45 years; p < 0.001) and had significantly longer back pain duration (11.36 ± 7.73 vs. 7.37 ± 2.99 months; p = 0.007) and leg pain duration (9.50 ± 4.43 vs. 5.22 ± 1.48 months; p < 0.001). No significant differences were observed for sex (p = 0.365), BMI (p = 0.197), diabetes mellitus (p = 0.806), hypertension (p = 0.135), osteoporosis (p = 0.857), education, or occupation. Variables with p < 0.20 on bivariate screening - age, back pain duration, leg pain duration, hypertension, and BMI - were entered into a binary logistic regression model with backward stepwise elimination (removal criterion p > 0.10). Two factors independently associated with TSS were identified in the final model (Table 4): older age (adjusted odds ratio (aOR) 1.06, 95% CI: 1.03-1.08; p < 0.001) and longer leg pain duration (aOR 1.13, 95% CI: 1.04-1.23; p = 0.004). Back pain duration, hypertension, and BMI were eliminated (Table 4).

**Table 3:** Bivariate Comparison of Demographic and Clinical Variables by TSS Status Continuous variables were compared using Student's independent samples t-test; categorical variables were compared using Pearson's chi-squared test. *p < 0.05. **p < 0.01. ***p < 0.001. TSS: tandem spinal stenosis; BMI: body mass index; SD: standard deviation; t: Student's independent samples t-test statistic; χ²: Pearson's chi-squared test statistic

Variable	TSS-positive (n = 181)	TSS-negative (n = 45)	Test statistic	p-value
Demographics				
Age (years), mean ± SD	58.29 ± 12.77	48.40 ± 15.45	t = 4.48	<0.001***
Age group, n (%)			χ² = 12.87	<0.001***
<50 years	45 (24.9%)	23 (51.1%)		
50-65 years	78 (43.1%)	14 (31.1%)		
>65 years	58 (32.0%)	8 (17.8%)		
Sex, n (%)			χ² = 0.82	0.365
Female	95 (52.5%)	27 (60.0%)		
Male	86 (47.5%)	18 (40.0%)		
Education level, n (%)			χ² = 8.16	0.043*
No formal education	7 (3.9%)	1 (2.2%)		
Primary school	34 (18.8%)	8 (17.8%)		
Secondary school	79 (43.6%)	11 (24.4%)		
College/university	61 (33.7%)	25 (55.6%)		
Occupation, n (%)			χ² = 1.42	0.701
Office work	42 (23.2%)	11 (24.4%)		
Manual labor	41 (22.7%)	8 (17.8%)		
Unemployed	59 (32.6%)	13 (28.9%)		
Retired	39 (21.5%)	13 (28.9%)		
Anthropometrics				
Weight (kg), mean ± SD	87.73 ± 11.63	89.89 ± 10.95	t = -1.12	0.263
Height (m), mean ± SD	1.72 ± 0.08	1.71 ± 0.08	t = 0.75	0.452
BMI (kg/m²), mean ± SD	29.92 ± 5.02	30.99 ± 4.83	t = -1.29	0.197
BMI category, n (%)			χ² = 0.26	0.876
Normal	23 (12.7%)	6 (13.3%)		
Overweight	67 (37.0%)	16 (35.6%)		
Obese	89 (49.2%)	22 (48.9%)		
Clinical symptoms				
Back pain present, n (%)	111 (61.3%)	29 (64.4%)	χ² = 0.15	0.700
Back pain duration (months), mean ± SD	11.36 ± 7.73	7.37 ± 2.99	t = 2.73	0.007**
Neck pain present, n (%)	58 (32.0%)	12 (26.7%)	χ² = 0.49	0.483
Neck pain duration (months), mean ± SD	11.23 ± 8.12	9.87 ± 6.94	t = 0.91	0.182
Leg pain present, n (%)	92 (50.8%)	19 (42.2%)	χ² = 1.07	0.302
Leg pain duration (months), mean ± SD	9.50 ± 4.43	5.22 ± 1.48	t = 4.25	<0.001***
Arm pain present, n (%)	76 (42.0%)	17 (37.8%)	χ² = 0.26	0.610
Arm pain duration (months), mean ± SD	7.82 ± 4.05	7.28 ± 3.37	t = 0.53	0.599
Comorbidities, n (%)				
Diabetes mellitus	72 (39.8%)	17 (37.8%)	χ² = 0.06	0.806
Hypertension	34 (18.8%)	13 (28.9%)	χ² = 2.23	0.135
Osteoporosis	38 (21.0%)	10 (22.2%)	χ² = 0.03	0.857
Rheumatological disorders	50 (27.6%)	6 (13.3%)	χ² = 3.98	0.073
Tumors	12 (6.6%)	2 (4.4%)	χ² = 0.11	0.742
Other diagnoses	23 (12.7%)	4 (8.9%)	χ² = 0.20	0.653

**Table 4:** Binary Logistic Regression - Crude and Adjusted Predictors of TSS *p < 0.05. **p < 0.01. ***p < 0.001. OR: odds ratio; CI: confidence interval; Ref.: reference category; TSS: tandem spinal stenosis; BMI: body mass index

Variable	Category	Crude OR (95% CI)	p-value	Adjusted OR (95% CI)	p-value
Age	Per 1-year increase	1.06 (1.03-1.08)	<0.001***	1.06 (1.03-1.08)	<0.001***
Leg pain duration	Per 1-month increase	1.13 (1.04-1.23)	0.003**	1.13 (1.04-1.23)	0.004**
Back pain duration	Per 1-month increase	1.04 (0.99-1.10)	0.085	-	-
Hypertension	Yes vs. no	0.57 (0.27-1.20)	0.138	-	-
BMI	Per 1 kg/m^2^ increase	0.96 (0.90-1.02)	0.197	-	-
Sex (female)	Female vs. male	0.74 (0.38-1.43)	0.366	-	-
Diabetes mellitus	Yes vs. no	1.09 (0.56-2.13)	0.806	-	-
Osteoporosis	Yes vs. no	0.93 (0.42-2.05)	0.857	-	-

## Discussion

This study reports an 80.1% prevalence of TSS (Lee Grade ≥ 1) among 226 patients presenting with symptomatic spinal stenosis at a tertiary neurosurgical center, which is at the higher end of the 7.6%-60% range reported in the literature [22]. This elevated prevalence likely reflects both our clinical sampling frame, as patients referred for specialist neurosurgical assessment represent a pre-selected, more severely affected population, and the inclusive radiological definition (Grade ≥ 1) adopted in the primary analysis. The Wakayama Spine Study, which required moderate-to-severe stenosis, reported an 11% prevalence in the general population [10]. Meanwhile, studies restricted to patients with pre-existing stenosis report rates of 32%-85% [23]. Notably, our sensitivity analysis using Grade ≥ 2 (moderate-to-severe) yielded a substantially lower prevalence of 45.6% (95% CI: 39.1%-52.1%), demonstrating the considerable impact of the stenosis severity threshold on prevalence estimates. The absence of a universally accepted radiological definition of TSS remains a major source of heterogeneity in prevalence data across the literature [5,12].

CLTSS was the overwhelmingly dominant subtype (97.2%), consistent with the established epidemiology of TSS, in which the cervical and lumbar regions are most frequently coaffected due to shared etiological degenerative mechanisms, including spondylosis, disc degeneration, and LFH [12]. The near-complete absence of thoracic involvement (2.2%) aligns with reports that thoracic stenosis is the rarest manifestation, often attributable to OLF, a pathology more prevalent in East Asian populations [9]. However, recent large-cohort data show that when degenerative thoracic stenosis is present, the likelihood of concomitant tandem lesions is exceptionally high, reaching up to 42%, underscoring the need to maintain a high index of clinical suspicion for remote spinal lesions if any thoracic involvement is detected [24].

Age emerged as the factor most strongly associated with TSS (aOR 1.06 per year; p < 0.001), consistent with the well-established role of advancing age in driving multi-level degenerative spinal disease. As the global population ages, the burden of TSS is expected to rise substantially [11]. The association between longer leg pain duration and TSS (aOR 1.13; p = 0.004) suggests that prolonged lower-limb neurological symptoms may serve as an early clinical indicator of multi-regional stenotic disease, supporting an argument for earlier whole-spine imaging in this group.

Notably, neither hypertension (p = 0.135), BMI (p = 0.197), nor diabetes mellitus (p = 0.806) was significantly associated with TSS on bivariate or multivariate analysis. Although hypertension and BMI met the initial screening threshold (p < 0.20) for entry into the multivariable model, both were eliminated, suggesting that their crude associations with TSS are likely confounded by age. The absence of a significant association between BMI and TSS contrasts with some reports suggesting obesity as a risk factor for degenerative spinal disease, though its role in multi-level stenosis specifically remains debated [1].

The diagnostic challenge of TSS is well recognized. The characteristic blunting of upper motor neuron signs by coexisting lumbar lower motor neuron pathology means that a substantial proportion of TSS cases may be missed on clinical examination alone [25,26]. Indeed, retrospective data demonstrate that up to 45% of TSS cases suffer a delayed or missed diagnosis, often necessitating secondary decompressive surgeries months after the initial procedure due to persistent or newly unmasked symptoms [19]. Given this high risk of delayed diagnosis, our findings suggest that a low threshold for whole-spine MRI may be clinically warranted, particularly in patients over 50 years presenting with chronic leg pain, to help avert missed diagnoses and facilitate appropriately planned surgical intervention.

Limitations

Several limitations should be acknowledged. The single-center design may limit generalizability to other populations. The cross-sectional design precludes causal inference. Symptomatic assessment was patient-reported and not systematically scored using validated instruments (e.g., mJOA and Nurick Scale), which may have introduced ascertainment bias. Finally, residual confounding from unmeasured variables cannot be excluded despite multivariable adjustment. Future multi-center longitudinal studies with validated functional outcome measures are warranted.

## Conclusions

This study demonstrates a high prevalence of TSS (80.1% using Lee Grade ≥ 1; 45.6% using Grade ≥ 2) among 226 patients presenting with symptomatic spinal stenosis at a tertiary neurosurgical center, with cervico-lumbar involvement being the dominant subtype (97.2%). Advancing age and prolonged leg pain duration are independently associated with TSS. The substantial difference between primary and sensitivity prevalence estimates highlights the importance of standardizing TSS definitions in future research. These findings suggest that a low threshold for whole-spine MRI may be clinically warranted in older patients with chronic radicular symptoms to avoid missed diagnoses and enable timely, appropriately planned surgical intervention.
